# Case series: Primary aldosteronism diagnosed despite normal screening investigations: A report of three cases

**DOI:** 10.1097/MD.0000000000033724

**Published:** 2023-05-17

**Authors:** Minyue Jia, Liya Lin, Hanxiao Yu, Boyun Yang, Xiaohong Xu, Xiaoxiao Song

**Affiliations:** a Department of Ultrasonography, The Second Affiliated Hospital Zhejiang University School of Medicine, Hangzhou, China; b Clinical Research Center, The Second Affiliated Hospital Zhejiang University School of Medicine, Hangzhou, China; c Department of Allergy, The Second Affiliated Hospital Zhejiang University School of Medicine, Hangzhou, China; d Department of Endocrinology, The Second Affiliated Hospital Zhejiang University School of Medicine, Hangzhou, China.

**Keywords:** adrenal venous blood sampling, aldosterone-to-renin ratio, case report, obstructive sleep apnea, primary aldosteronism, renin

## Abstract

Since the introduction of the aldosterone-to-renin ratio (ARR) as a screening tool for primary aldosteronism (PA), there has been a marked increase in the reported prevalence of this condition among hypertensive, even normotensive, subjects.

**Patient concerns:**

But ARR as a spot blood draw for estimating a patient’s aldosterone secretory status is influenced by many factors.

**Diagnoses:**

Here, we describe a series of patients with biochemically confirmed PA, whose diagnosis was delayed by the initial ARR assessment with non-suppressed renin.

**Interventions:**

Patient 1 had a history of resistant hypertension for many years and had a negative initial screening for secondary hypertension (including ARR). At the reevaluation, ARR was close to cutoff still with normal renin after strict and extended drug washout, and the further workup for PA demonstrated a unilateral aldosterone producing adenoma that was surgically removed, with subsequent complete biochemical remission and partial clinical success. Patient 2 was diagnosed with idiopathic hyperaldosteronism combined with obstructive sleep apnea syndrome, which could increase renin resulting in a negative ARR, and finally got a better treatment effect with PA-specific spironolactone, as well as continuous positive airway pressure. Patient 3 with hypokalemia as the main presentation was finally diagnosed with PA after excluding other diseases, and proceeded to laparoscopic adrenalectomy and histologically confirmed an aldosterone producing adenoma. Postoperatively, patient 3 achieved complete biochemical success without any medicine.

**Outcomes:**

The clinical status of all three patients was effectively managed, resulting in either complete resolution or notable improvement of their respective conditions.

**Lessons:**

After rigorous standardized diagnostic evaluation, there are still many reasons for ARR negative in PA, but they all basically occur in the background of normal or normal-high renin without suppression. A negative screening test result should be repeated and analyzed carefully if this is not consistent with the clinical picture. If, despite a repeatedly negative ARR, clinical suspicion remains high, we recommend consideration of further evaluation, including confirmatory tests and adrenal venous blood sampling (AVS) or even 68Ga-pentixafor PET/CT to better confirm the diagnosis and improve patient outcomes.

## 1. Introduction

Primary aldosteronism (PA) is the commonest and most modifiable form of secondary hypertension, which occurs due to abnormal excessive aldosterone production from the adrenals. Aldosterone-to-renin ratio (ARR) is the most reliable screening method of PA and has been widely used in clinical practice, but the index is influenced by many factors, some of which cause it negative, consequently leading to PA underdiagnosed. We report 3 cases of biochemically confirmed PA, where initial screening and biochemical tests were potentially misleading. These cases highlight the importance of clinical suspicion in the current diagnostic approach to PA.

## 2. Case presentation

### 2.1. Patient 1

**Figure 1.**
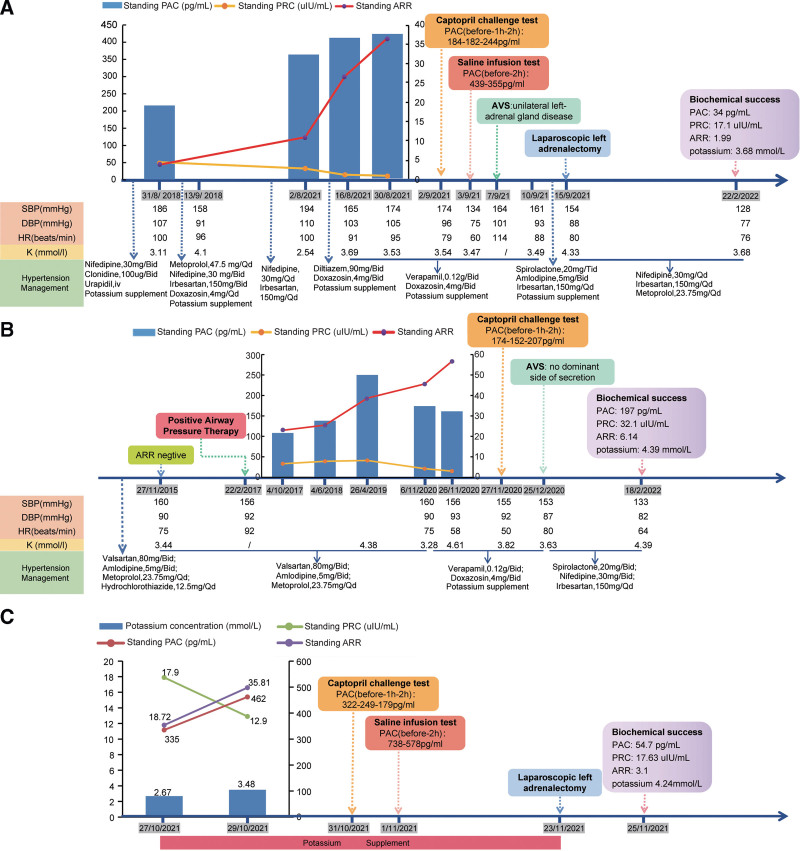
Clinical timeline of 3 cases. (A) timeline of Patient 1; (B) timeline of Patient 2; (3) timeline of Patient 3. ARR = aldosterone-to-renin ratio, PAC = plasma aldosterone concentration, PRA = plasma renin activity.

A 45-year-old adipose male (height 164 cm, weight 76.8 kg) had elevated blood pressure for 10 years, and the highest blood pressure was 234/123 mm Hg. In 2018, he was hospitalized for poor blood pressure control combined with dizziness, even with multiple antihypertensive drugs (see timeline, Fig. 1A). The diagnostic tests performed at that time revealed the following: low serum potassium, kaliuresis, proteinuria (qualitative 1+), normal plasma aldosterone concentration (PAC), slightly elevated plasma renin concentration (PRC) and normal ARR (Table 1). A computed tomography (CT) scan of the abdomen revealed a 12.6 × 12.9 mm left adrenal nodule. Secondary hypertension was ruled out by laboratory and imaging examinations. A diagnosis of essential hypertension and left adrenal nonfunctional adenoma was reached. Treatment included metoprolol, nifedipine, irbesartan, doxazosin and potassium supplementation and then the patient was discharged. In the following years, the patient referred only the primary care physician, and blood pressure remained poorly controlled.

**Table 1:** Biochemical summary of the patient 1.

Biochemical data	In 2018	Before surgery in 2021	Postoperative in 2022	Normal range
Serum levels of electrolytes				
Na (mmol/L)	141.2	141.7	138.8	135.0–145.0
K (mmol/L)	3.11↓	2.54↓	3.68	3.50–5.50
Cl (mmol/L)	101.9	99.7	100.9	96.0–106.0
Urinary K (mmol/24 h)	30.80	41.80	32.49	25.0–100.0
eGFR mL/(min·1.73 m^2^)	85.15↓	88.09↓	73.11↓	≥90
Urine microalbumin (mg/g.Cr)	27.01↑	40.34↑	10.11	<25.0
Fasting plasma glucose (mmol/L)	6.16↑	7.83↑	5.08	3.89–6.11
HbA1c (%)	6.4↑	7.4↑	6.3↑	4.0–6.0
Triglycerides (mmol/L)	3.02↑	3.65↑	2.66↑	<1.7
Standing PAC (pg/mL)	220	367↑	34	30.0–353.0
Standing PRC (uIU/mL)	51.5↑	33.4	17.1	4.4–46.1
Standing ARR	4.27	10.99	1.99	<37
Heart echocardiography	Left ventricular hypertrophy	Left ventricular hypertrophy	Normal	

ARR = aldosterone renin ratio, PAC = plasma aldosterone concentration, PRC = plasma renin concentration.

**Figure 2.**
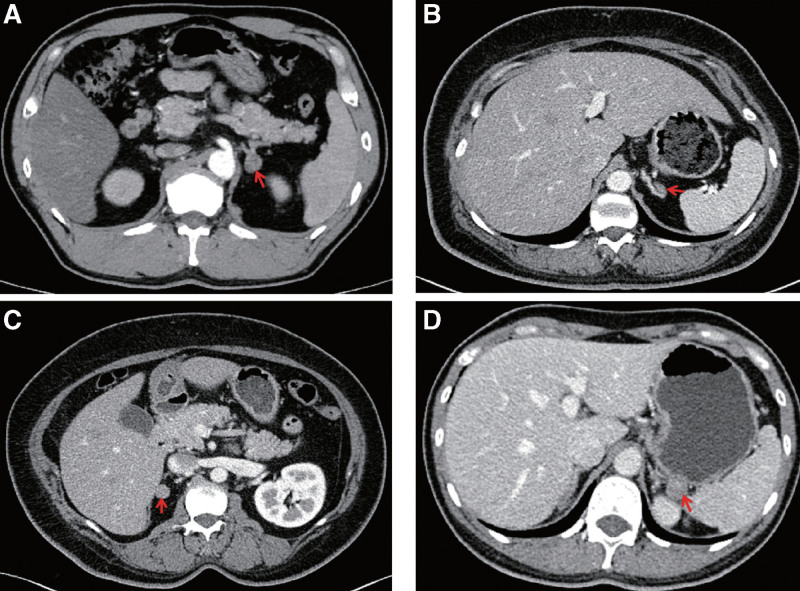
Results of adrenal contrast-enhanced CT of the 3 patients: (A) adrenal computed tomography enhancement in patient 1 indicating mild to moderate enhancement of left adrenal adenoma (pointed by the red arrow). (B) and (C) adrenal computed tomography enhancement in patient 2 shows heterogeneous enhancement of bilateral adrenal nodules (pointed by red arrows). (D) adrenal computed tomography enhancement in patient 3 indicating marked persistent enhancement of the left adrenal adenoma (pointed by the red arrow).

**Figure 3.**
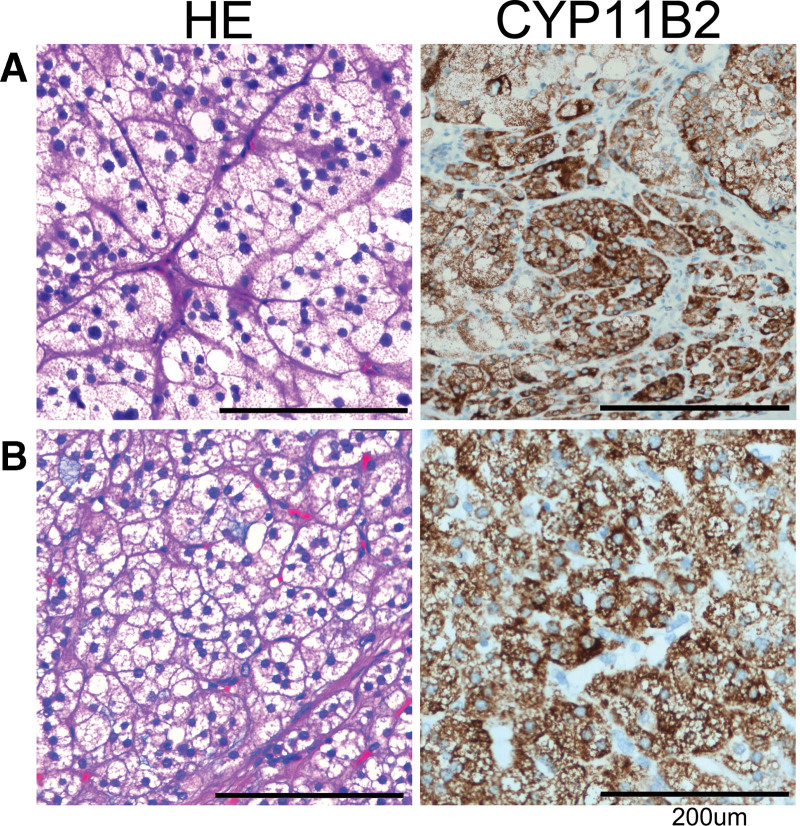
Immunohistochemistry of adrenal adenoma. (A) Hematoxylin & eosin staining and CYP11B2 immunohistochemistry of patient 1; (B) Hematoxylin & eosin staining and CYP11B2 immunohistochemistry of patient 3.

Upon the patient’s hospitalization again 3 years later for the severe hypertension (194/110 mm Hg) and hypokalemia (2.54 mmol/L, reference range: 3.50–5.50 mmol/L), despite oral administration of nifedipine and irbesartan. Auxiliary examination results showed: hypokalemia, kaliuresis, metabolic syndrome, mild renal dysfunction, and proteinuria (24-hour urine protein quantification was 361 mg/24 h, reference range: 28–141 mg/24 h) (Table 1). A 1mg low-dose dexamethasone suppression test was negative. And ARR screening for PA was still negative, with slightly elevated PAC (367 pg/mL, reference range: 30.0–353.0 pg/mL), normal PRC (33.4 uIU/mL, reference range: 4.4–46.1 uIU/mL), and normal ARR (10.99, reference range: <37). No abnormality was found on renal artery duplex ultrasonography and abdominal CT angiography as well as full-night standard polysomnography. Echocardiography presented left ventricular hypertrophy (LVH, interventricular septum-12.3 mm, left ventricle-40.7 mm, left atrium-34.4 mm). Considering the impact of current antihypertensive therapy on ARR screening, diltiazem, and doxazosin, were instead, and potassium supplementation was also given at the same time. After 2 weeks, PAC increased (415 pg/mL), PRC decreased (15.6 uIU/mL), and the corresponding ARR (26.6) increased, but the cutoff value for ARR positivity was still not reached because of high levels of renin. At the same time, diltiazem was adjusted to verapamil, 0.12 g/bid due to poor blood pressure control. After another 2 weeks, ARR was tested again, and it was found that the ARR was significantly increased (PAC: 424 pg/mL, PRC: 11.6 uIU/mL, ARR: 36.55, potassium: 3.53 mmol/L) (see timeline, Fig. 1A). Moreover, the PAC was not sufficiently suppressed both on the captopril challenge test and saline infusion test, which confirmed a diagnosis of PA. The adrenal CT contrast-enhanced scan showed a nodular low-density shadow on the left adrenal gland, approximately 18 mm in size (slightly larger than 2018), with mild to moderate enhancement on contrast injection (Fig. 2A). Adrenal venous sampling (AVS) revealed an aldosterone/cortisol ratio of the left adrenal vein that was approximately 10 times greater than on the right side, which definitively confirmed unilateral left-sided disease. Collectively, we diagnosed the patient with PA due to a left-sided aldosterone-producing adenoma (APA). The patient then underwent laparoscopic left adrenalectomy. The pathologic examination revealed a 15 mm APA (Fig. 3A). Postoperatively, patients achieved complete biochemical success, but still needed 3 kinds of antihypertensive drugs to control blood pressure. In addition, 6 months after the operation, echocardiography showed that the heart had returned to normal (left ventricular mass indexed to body surface area, from 93.46 to 72.17 g/m^2^). However, his renal function did not improve significantly after surgery (glomerular filtration rate from 88.09 mL/[min·1.73 m^2^] to 73.11 mL/[min·1.73 m^2^]).

### 2.2. Patient 2

A 40-year-old woman with a history of metabolic syndrome was referred to endocrinology clinic for the evaluation of resistant hypertension. In 2015, she was found to have a high blood pressure of 200/140 mm Hg when she visited a doctor for “nosebleed.” A routine clinical evaluation highlighted elevated blood pressure values even with multiple antihypertensive drugs so that, considered the young age of the patient (35 years old), a panel of laboratory and instrumental tests was performed in order to exclude secondary hypertensive forms and to evaluate the associated cardiovascular burden. At that time, she was obese, body mass index (BMI): 30.67 kg/m^2^, waist circumference 97 cm, hip circumference 110 cm. Ambulatory blood pressure monitoring indicated that the blood pressure was higher than normal (highest systolic blood pressure 167 mm Hg, highest diastolic blood pressure 119 mm Hg). Echocardiography presented left heart enlargement (interventricular septum-8.3 mm, left ventricle-54.3 mm, left atrium-47.4 mm). Auxiliary examination results showed: hypokalemia (potassium: 3.44 mmol/L), kaliuresis (24 h urinary potassium 58.3 mmol/24 h), and metabolic syndrome. Urinary vanillylmandelic acid, thyroid function, and urine routine were in the normal range. A 1 mg low-dose dexamethasone suppression test was negative. ARR screening for PA was negative: ARR was 125 (reference range: <300) in upright position (PAC: 87.5 pg/mL, reference range: 70–300 pg/mL, plasma renin activity: 0.70 ng/mL/h, reference range: 0.1–6.56 ng/mL/h). Enhanced CT scan of the adrenal glands revealed a nodule with a diameter of about 9 mm in each adrenal gland. There was no evidence either of kidney disease or of renovascular disease. The final diagnosis was “essential hypertension with initial organ damage” and the patient started treatment with valsartan, amlodipine and metoprolol (see timeline, Fig. 1B).

In 2017, the patient presented to the doctor for nighttime sleep snoring. Full-night polysomnography indicated a large number of apnea and hypopnea during sleep, the AHI was 74 events/h, and the minimum nighttime blood oxygen saturation was 20%. Therefore, severe obstructive sleep apnea syndrome (OSA) combined with severe nocturnal hypoxemia was diagnosed. Together with lifestyle recommendations, continuous positive airway pressure (CPAP) therapy was applied and a pressure of 13 cm H_2_O was administered after titration. But the patient’s blood pressure still fluctuated over the following years. At the same time, aldosterone was regularly reviewed, and it was found that with the treatment of CPAP, the patient’s renin level showed a downward trend and the ARR increased accordingly under the same antihypertensive medication (see timeline, Fig. 1B).

In 2020, the patient referred to our endocrinology department because of a poor blood pressure control. After a careful clinical reevaluation and a critical reappraisal of previous data, we decided to repeat a complete screening for secondary hypertension, being evident a systo-diastolic hypertension, with elevated blood pressure (140/90 mm Hg in the morning and 160/90 mm Hg in the afternoon). She had adequate physical development, never smoke or drink, had normal salt intake in the diet, denied any use of drugs that induce hypertension, including VEGF inhibitors, calcineurin inhibitors, NSAIDs, glucocorticoids, and oral contraceptive pills. At physical examination, we observed a BMI of 33.46 kg/m^2^, a waist circumference of 107 cm, a hip circumference of 118 cm and a neck circumference of 46 cm. There were no alterations of ACTH or cortisol concentrations, urinary metabolites of catecholamines and glucocorticoid hormones, but serum potassium was reduced (3.28 mmol/L) and ARR was clearly increased (ARR: 53.67, PAC: 161 pg/mL, PRC: 3.0 uIU/mL). The hormonal determinations were performed after the substitution of potentially interfering drugs with verapamil and doxazosin. During the saline infusion test, the patient’s blood pressure increased significantly, so captopril challenge test was instead in which the biochemical diagnosis of PA was confirmed (PAC 207 pg/mL after testing) (see timeline, Fig. 1B). Adrenal contrast-enhanced CT identified that the size of bilateral adrenal nodules increased slightly, from 9 to 11 mm on both sides (Fig. 2B and C). In addition, echocardiography proved an increase of the previous finding of LVH (left ventricular mass indexed to body surface area from 85.07 to 140.99 g/m^2^). Then AVS led to the demonstration of no dominant side of secretion (LI: 1.85, CI: 1.19). Ultimately, the patient was diagnosed as idiopathic hyperaldosteronism. The treatment included spirolactone, nifedipine, irbesartan, as well as CPAP. Until now, the patient feels completely well and is normotensive with normal serum potassium and ARR. Furthermore, the patient’s metabolic indicators including fasting insulin, and low-density lipoprotein cholesterol were also significantly improved (fasting insulin dropped from 204.6 pmol/L in 2019 to 79.1 pmol/L in 2022; low-density lipoprotein cholesterol dropped from 3.06 mmol/L in 2019 to 1.46 mmol/L in 2022).

### 2.3. Patient 3

A 35-year-old married woman was hospitalized due to lower extremity weakness for 1 month. The local hospital emergency checked the serum potassium 2.65 mmol/L, and the symptoms improved following potassium supplementation. Afterward, the patient repeatedly experienced fatigue, which was accompanied by nocturia (6–7 times/night), and without associated symptoms of fever, night sweats, diarrhea, vomiting, or dizziness, and no record of hypertension during this period. The patient reported no previous use of drugs, including diuretics, licorice, herbal supplements, antibiotics and steroids, and had no history of hypertension, diabetes, thyroid disease and urinary stones. There was also no relevant family history.

On physical examination, clinical and vital parameters (height 161 cm; weight 47.6 kg; pulse 97 beats/min; and blood pressure 125/79 mm Hg) were normal. The patient reported that the blood pressure was normal at ordinary times, but occasionally had high-normal blood pressure. The 24-hour ambulatory blood pressure monitoring was recommended, but the patient refused. The results of initial investigations, including a complete blood count, liver function tests, and electrocardiogram, were all within the reference range. Chest CT unexpectedly found a left adrenal nodule. Blood biochemistry showed that the patient had persistent hypokalemia (range 2.65–3.48 mmol/L; the latter value was recorded after potassium supplementation), associated with kaliuresis (24 hours urinary potassium 86.38 mmol/24 h, transtubular potassium gradient, 7.61). The result of arterial blood gas was normal (pH 7.417, reference range: 7.350–7.450, plasma bicarbonate 25.4 mmol/L, reference range: 22.0–26.0 mmol/L). The levels of other electrolytes (including sodium, chloride, calcium, magnesium, phosphorus), thyroid hormones, serum lipid profile, 24-hour urinary free cortisol, 24-hour urinary vanilla mandelic acid, and glycated hemoglobin were all normal. Routine urinalysis was also normal (urine pH 7.0). Renal artery duplex ultrasonography showed no abnormality. There was also no evidence of kidney disease and systemic rheumatic disease. Given the patient’s spontaneous hypokalemia with adrenal incidentaloma, PA was still suspected despite normotensive. The PAC and PRC in the standing position were within normal limits, giving an ARR of 18.72 (PAC: 335 pg/mL, PRC: 17.9 uIU/mL, potassium: 2.67 mmol/L) on day 2 after admission (see timeline, Fig. 1C). After 2 days of potassium supplementation, the aldosterone and renin concentrations were tested again, and it was found that the ARR increased significantly but renin was still not inhibited (PAC: 462 pg/mL, PRC: 12.9 uIU/mL, ARR: 35.81, potassium: 3.48 mmol/L). Meanwhile, her parathyroid hormone was elevated (129.90 pg/mL, reference range: 15.00–65.00 pg/mL). As the ARR was very closed to 37, thus a further captopril challenge test was still carried out, and the results showed that PAC after 2 hours was higher than 110 pg/mL. Moreover, during the intravenous infusion of 2 L of 0.9% saline over 4 hours in supine position, there was no adequate suppression of PAC on volume expansion. Thus, the above-mentioned results confirmed a diagnosis of PA. Adrenal contrast-enhanced CT identified a 13 × 15 mm left adrenal solid mass with typical features of an adenoma (Fig. 2D).

The patient underwent laparoscopic left adrenalectomy without having an AVS study. This is in agreement with the current guideline because our patient was aged less than 35 years, and the unilateral nodule size was above 1 cm at CT.^[1]^ Meanwhile, the patient also refused to do AVS. Postoperative histopathology confirmed an aldosterone producing adenoma (Fig. 3B). Postoperatively, the patient achieved complete biochemical success as well as normal parathyroid hormone. Blood pressure values on repeated measurements were persistently less than 140/90 mm Hg without any medicine.

## 3. Discussion and conclusions

PA was first reported by Jerome W. Conn in 1954 and was considered a rare disorder, only suspected in cases with hypertension and spontaneous hypokalemia. In recent years, with the widespread use of ARR as a screening test, the prevalence of PA has increased from 0.7% to 29.8%, depending on the population selected and the diagnostic values used.^[2]^ PA as a curable or controllable clinical disease, it is important to identify such patients at early stage because they have a higher morbidity and mortality from cardiovascular disease than age and sex matched patients with essential hypertension.^[3]^ According to the current guidelines, PA can be diagnosed by a positive orthostatic ARR and confirmatory tests, but many factors affect the results.^[4]^ Common factors causing negative ARR include potassium status, sodium loading, pregnancy, antihypertensive drugs, some complicating diseases such as OSA and renal insufficiency and some undiscovered causes.^[3,5–12]^ These negative ARRs may have different causes, but they all generally occur in the context of normal or normal-high renin. As the ARR is strongly influenced by the PRC as the denominator of the ratio such that minor changes in renin may lead to considerable changes in the ARR. Active renin secretion is regulated principally by 4 interdependent factors: pressure in the afferent arteriole, Na+ at the macula densa, sympathetic nerve stimulation via beta-1 adrenergic receptors, and negative feedback by Ang II.^[13]^ Any factor or disease that can cause elevated renin may result in a negative ARR. It has been reported that approximately 27.5% to 29% of PA patients can present with normal or even elevated renin levels, which could influence diagnostic accuracy of ARR.^[14,15]^ In fact, it is also unknown what proportion of PA patients could have a non-suppressed renin as patients with a normal screening test would normally not be referred for further confirmatory testing. Therefore, in the context of screening for or confirming biochemical hyperaldosteronism, normal results should not be considered totally definitive. In the case of high clinical suspicion of PA but negative ARR with normal or normal-high renin, even after controlling known confounders of the ARR such as posture, dietary sodium intake, potassium status, timing of the menstrual cycle, and influence of antihypertensive medications, the diagnoses could be directly entered into the confirmation tests even the repeated ARR is still normal.

Patient 1 still had high blood pressure under the 3 kinds of antihypertensive drugs including an intravenous drip of urapidil, and the initial screening for PA was negative. Nifedipine as a dihydropyridine calcium antagonist and irbesartan as an angiotensin receptor blocker can increase renin production and reduce aldosterone, thereby potentially confounding the biochemical assessment of PA. The patient’s ARR increased after 2 weeks of drug washout and approached the positive cutoff after 4 weeks of washout. However, after strict drug washout at this time, the renin level was still 11.6 uIU/mL, which was greater than 8.4 uIU/mL. After rigorous standardized diagnostic evaluation, so what could have caused his renin not to be inhibited? It is worth noting that this patient had high urinary protein quantification, abnormal urinary renal function, and foam urine for several years, so his renal function has been impaired to some extent. Chronic kidney disease (CKD) activates the renin-angiotensin-aldosterone system (RAAS), resulting in a rise in renin. This may contribute to the underdiagnosis of PA in CKD patients, which is consistent with recent findings.^[16,17]^ Meanwhile, plasma renin was in a normal/high-normal range, suggesting underlying intrarenal vascular damage causing glomerular ischemia and renin escape from suppression by aldosterone excess.^[15]^ In addition, recent studies have found that higher pretreatment plasma renin levels predict lower percentage of cure of aldosteronism and greater need for antihypertensive agents to reach adequate control of blood pressure.^[18]^ Just as our patient 1 achieved complete biochemical success but still needed 3 antihypertensive drugs after adrenal surgery. Indeed, hypertension may persist after adequate treatment of aldosteronism, and about 61% require persistent use of one or more antihypertensive agents after unilateral adrenalectomy.^[4]^ The blood pressure outcome of surgery is not only dependent on normalization of aldosterone secretion, but also on the existing deleterious vascular and renal changes determined by the duration of the preceding aldosterone-induced hypertension, as well as age, gender, BMI and other comorbidities.^[19,20]^ Our Patient 1, male, had a long course of hypertension (10 years), also combined with metabolic syndrome and mild renal insufficiency, as well as relatively high renin concentration before treatment. All these factors may lead to the incomplete resolution of hypertension after adrenalectomy. Therefore, timely identification of PA is important to maximize the benefits of treatment.

Patient 2 was a refractory hypertensive patient with PA and OSA. In the first evaluation the screening test was negative, with ARR value far below the limit. These considerations induced the clinicians who firstly evaluated the patient to stop further investigations. Intermittent hypoxia of severe OSA coexisting in this patient activated the RAAS and sympathetic nervous systems, resulting in an increase in PRC and a negative ARR.^[21,22]^ After CPAP for OSA, the renin gradually decreased, resulting in a gradual increase in the ARR, while antihypertensive drugs remained unchanged. But this process took 3 years. During the period of CPAP, the apnea during sleep was much less than before (AHI from 74 to 14.8 events/h), indicating that the CPAP treatment was effective. Therefore, the effect of CPAP therapy for OSA on the renal RAAS could be a long and complex process. Research has found that the prevalence of OSA among patients with PA is reportedly as high as 67%, but the rates of PA in unselected patients with OSA are lower.^[23,24]^ PA patients were not detected in OSA patients because OSA-induced ARR negatives prevented further diagnosis of PA, and its prevalence is merely underestimated.^[25]^ Furthermore, the coexistence of both PA and OSA led to the progressive development of LVH in the patient 2. Although it might be postulated that the inappropriate sodium and fluid retention characterizing hyperaldosteronism might contribute to the development of LVH, other factors such as the co-existence of OSA may determine the LV geometry.^[26]^ As both OSA and hyperaldosteronism lead to the increased cardiovascular morbidity and mortality, the 2 conditions require prompt diagnoses and treatment. PA-directed therapy leads to an improvement in OSA severity. Similarly, CPAP treatment with good adherence can improve blood pressure with the downregulation of the renal RAAS activity in patients with OSA. Therefore, it is essential to screen all hypertensive patients affected by OSA for the presence of PA. It is worth noting that OSA affect the judgment of RAAS interference results when screening and diagnosing PA. For OSA patients whose ARR ratio does not reach the target, we recommend further confirmatory testing, because the decrease of renin in OSA may require a long treatment time. And further studies may be required to develop different ARR reference ranges for this subset of patients.

The normotensive Patient 3 initially presented with a negative ARR, partly presumedly due to severe and chronic hypokalemia. However, after potassium supplementation, her renin remained high (greater than 8.4 uIU/mL), resulting in an ARR approaching but not exceeding the positive threshold. It is notable that excessive sodium and insufficient potassium intake were important characteristics of Chinese dietary structure. The patient 3 had a 24-hour sodium excretion exceeding 100 mmol/d. Thus, dietary salt restriction was excluded that generally would lead to a rise in renin. Moreover, the patient was not pregnant, not in the menstrual period, had a normal renal function, and was in good health without other diseases. However, the resolution of hypokalemia after the removal of adrenal adenoma supported the major contribution of APA. A potential explanation for the escape of renin from inhibition is that unlike the complete inhibition of adrenocorticotropin by a cortisol-producing adenoma, some patients have inherently ineffective feedback mechanisms that result in a relative inability to completely inhibit renin release, and many factors such as ACTH and repeated orthostasis could also weaken or even reverse renin suppression and exhibit negative ARR.^[27,28]^ In addition, the blood pressure of this young PA patient has been always normal without any antihypertensive drugs. A normal ARR ratio combined with a normal blood pressure background is more likely to cause misdiagnosis and missed diagnosis. Although the ARR can be increased even in the absence of a suppressed plasma renin, this possibility should be kept in mind while performing the diagnostic workup of PA even in normotensive patients, and appropriate use of well-established confirmatory tests should be considered.^[29]^

**Figure 4.**
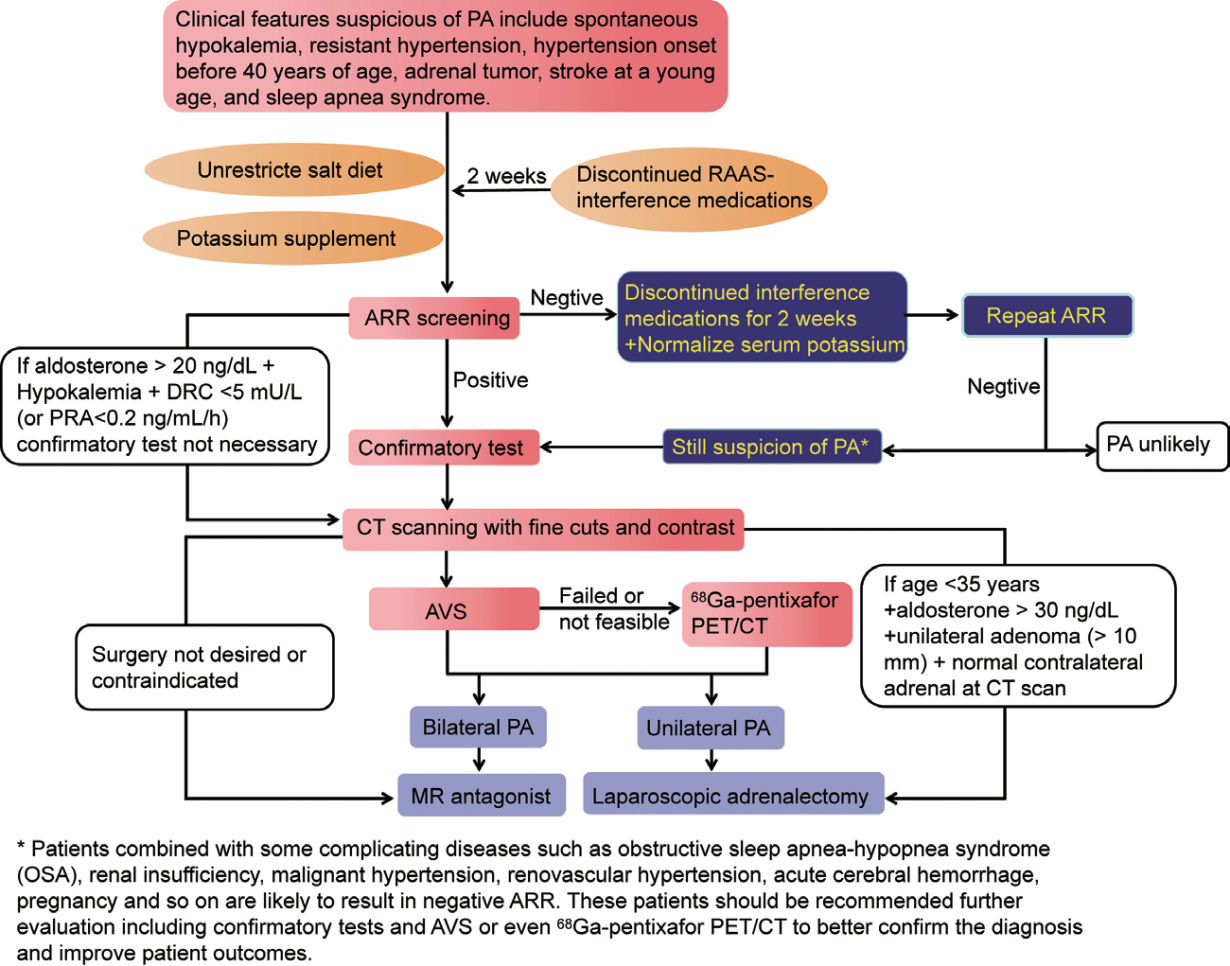
Proposed diagnostic flow-chart for patients with primary aldosteronism. ARR = aldosterone-to-renin ratio, AVS = adrenal venous blood sampling, PA = primary aldosteronism, PAC = plasma aldosterone concentration, PRA = plasma renin activity, RAAS = renin-angiotensin-aldosterone system.

Finally, we summarize some experience gained from these cases and some hints for future clinical work. It is important to recognize that PA patients with non-suppressed renin, hence a negative diagnosis ARR, exist.^[27]^ A single negative ARR influenced by many factors is insufficient for stopping the screening of PA. Negative ARR screening is usually caused by normal/high renin. After rigorous standardized diagnostic evaluation (Fig. 4), how to interpret ARR results should be carefully analyzed on a clinical basis. When there is a high clinical suspicion of PA, we recommend repeating ARR screening or considering further evaluation, including confirmatory testing and AVS or even ^68^Ga-pentixafor PET/CT,^[30]^ after all, surgical treatment or mineralocorticoid receptor antagonists may lead to significant improvements in blood pressure and cardiovascular disease and cerebrovascular events.

## Author contributions

**Conceptualization:** Minyue Jia, Xiaoxiao Song.

**Data curation:** Liya Lin, Hanxiao Yu, Boyun Yang.

**Methodology:** Xiaohong Xu.

**Writing – original draft:** Minyue Jia.

**Writing – review & editing:** Xiaoxiao Song.
